# Osteogenic and Chondrogenic Potential of the Supramolecular Aggregate T-LysYal®

**DOI:** 10.3389/fendo.2020.00285

**Published:** 2020-05-05

**Authors:** Adriana Di Benedetto, Francesca Posa, Mario Marazzi, Zamira Kalemaj, Roberta Grassi, Lorenzo Lo Muzio, Mariasevera Di Comite, Elisabetta Ada Cavalcanti-Adam, Felice Roberto Grassi, Giorgio Mori

**Affiliations:** ^1^Department of Clinical and Experimental Medicine, University of Foggia, Foggia, Italy; ^2^Department of Biophysical Chemistry, Heidelberg University & Max Planck Institute for Medical Research, Heidelberg, Germany; ^3^Struttura Semplice Tissue Therapy, Niguarda Hospital, Piazza dell'Ospedale Maggiore, Milan, Italy; ^4^Department of Basic and Medical Sciences, Neurosciences and Sense Organs, University of Bari, Bari, Italy; ^5^Department of Biomedical Sciences, University of Sassari, Sassari, Italy

**Keywords:** bone, cartilage, supramolecular aggregate, mesenchymal stem cells, tissue regeneration, focal adhesions

## Abstract

Hard tissue regeneration represents a challenge for the Regenerative Medicine and Mesenchymal stem cells (MSCs) could be a successful therapeutic strategy. T-LysYal® (T-Lys), a new derivative of Hyaluronic Acid (HA) possessing a superior stability, has already been proved efficient in repairing corneal epithelial cells damaged by dry conditions *in vitro*. We investigated the regenerative potential of T-Lys in the hard tissues bone and cartilage. We have previously demonstrated that cells isolated from the tooth germ, Dental Bud Stem Cells (DBSCs), differentiate into osteoblast-like cells, representing a promising source of MSCs for bone regeneration. Herewith, we show that T-Lys treatment stimulates the expression of typical osteoblastic markers, such as *Runx-2, Collagen I* (*Col1*) and Alkaline Phosphatase (ALP), determining a higher production of mineralized matrix nodules. In addition, we found that T-Lys treatment positively affects α_V_β_3_ integrin expression, key integrin in the osteoblastic commitment, leading to the formation of focal adhesions (FAs). The efficacy of T-Lys was also tested on chondrogenic differentiation starting from human articular chondrocytes (HACs) resulting in an increase of differentiation markers and cell number.

## Introduction

Tissue regeneration is one of the main research fields to be exploited in modern medicine.

Regenerative Medicine is an emerging branch combining different aspects of medicine, cell biology, and bioengineering to a common healing aim: to regenerate, to repair or replace tissues. Many scientists are testing new biological strategies to be used in both differentiated and stem cells to optimize tissue healing and replacement.

Bone regeneration based on Mesenchymal Stem Cells (MSCs) approach represents an innovative therapy of Regenerative Medicine. On the other hand, although cartilage differentiation from MSCs has been demonstrated, its clinical use is still occasional and with unsure predictability. Cartilage regeneration needs a different experimental procedure and can be obtained using methods as tissue grafts (autografts or allografts) or techniques adequate to stimulate the natural repair process (1).

The bone marrow still represents the gold standard to isolate MSCs. In order to overcome some adverse aspects connected with the harvest of MSCs from bone marrow, i.e., morbidity, pain, and concerns connected to the collection site, other sources of MSCs have been examined such as dental tissues. We have demonstrated that MSCs from dental bud *in vitro* differentiate effectively toward osteogenic lineage, representing a proper candidate for bone regeneration therapies (2, 3). However, the reconstruction of hard tissues defects, as bone and cartilage, still represents a challenge for regenerative medicine being the current treatments only partially effective and not always practicable. Natural molecules as Polydatin, Vitamin D or oxytocin are efficacious in driving the commitment of MSCs from dental bud to osteoblast, increasing the osteogenic differentiation and promoting the mineral matrix deposition (3–9). It has been demonstrated that extracellular matrix (ECM) plays an important role in cell differentiation of connective tissues (10, 11). MSCs interaction, via integrins, with ECM glycoproteins can enhance the osteogenic differentiation of these cells (3, 12). The ECM contains a varied set of macromolecules having not only roles in supporting the cells and determining the tissue structure, but also contributing to the growth factor diffusion and cell interactions with microenvironment, thus influencing the cell behavior. Glycosaminoglycans (GAGs) are complex ECM polysaccharides, with different chemical characteristics (i.e., sulfation, acetylation and epimerization), that confer a variety of functions such as: hydration, control of signaling molecules and enzymatic activity. Hyaluronic acid (HA) is a ubiquitous GAG, composed of repeating D-Glucuronic Acid and N-Acetyl-D-Glucosamine subunits, with a high molecular weight ranging between 0.2 and 10 million Daltons. Recently, a new derivative of HA has been developed; this molecule called T-LysYal® (T-Lys) is a supramolecular complex of HA combined with lysine hyaluronate, thymine, and sodium chloride. Due to the presence of such bounded groups, this innovative molecule forms longer tridimensional structures, compared to HA alone, known as nanotubes (13–16). Administration of T-Lys has shown promising results in tissue regeneration, improving the healing process of decubitus ulcers in humans (13), rapidly restoring nasal mucosa after functional endoscopic sinus surgery (15, 16) and repairing corneal epithelial cells damaged by dry conditions as demonstrated with the *in vitro* model of Dry Eye Syndrome (14). These results led us to speculate on a possible efficacy of T-Lys in bone and cartilage regeneration. To this purpose, in this study we evaluated *in vitro* the effects of T-Lys on osteogenic differentiation of MSCs from dental bud, analyzing the main molecular and histochemical markers of osteoblastogenesis. The chondrogenic differentiation from MSCs, requires different culture conditions and differentiation protocols which are still under investigation (17). Accordingly, for this study we used human articular chondrocytes (HACs). These cells maintained in bidimensional cultures, become flat shaped and secrete Collagen I rather than Collagen II. Therefore, to get and maintain their phenotype and functionality they need to be grown on three-dimensional systems (18). Consistently with this issue we studied T-Lys potentials on chondrogenic differentiation by using primary cultures of HACs grown in tridimensional pellets.

## Materials and Methods

### Ethics

The study was conducted in compliance with the Declaration of Helsinki and the International Conference on Harmonization Principles of Good Clinical Practice. The research protocol was approved by the ethical committee, within the project BIADIDEBT num. Rep 4159/2018, at the University of Foggia Ospedali Riuniti, and all participants gave informed consent allowing their anonymized information to be used for data analysis.

### Cell Cultures

#### Dental Bud Stem Cells (DBSCs) Cultures

Twenty healthy pediatric donors, sex matched and aged between 8 and 12 years, were selected for tooth bud extractions with piezo-surgery for orthodontic reasons, after the written informed consent was given from each patients' parents. The study was approved by the Institutional Review Board of the Department of Clinical and Experimental Medicine, University of Foggia. Once the buds were extracted, the tissues were dissected to remove the peripheral components corresponding to the enamel organ and to the dental follicle. The remaining internal part, the dental papillae, was enzymatically digested and filtered to obtain single-cell suspensions, harvested, seeded and expanded as previously described (3, 19–21). For osteogenic differentiation, cells were seeded at 3 × 10^3^/cm^2^ in α-MEM supplemented with 2% FBS and cell culture medium was supplemented with 10^−8^ M dexamethasone and 50 μg/ml ascorbic acid and with 10 mM β-glycerophosphate (Sigma Aldrich, Milan, Italy) for induction of matrix mineralization.

#### Chondrocyte Pellet Culture

Normal Articular Chondrocytes (HACs) were isolated from ten young patients (aged 19–38 years) subjected to knee arthroscopy for traumatic lesions, as described in Caron et al. (22). Dedifferentiated cells were seeded at 10 × 10^3^ cells/cm^2^ and cultured in DMEM High Glucose, 10% FBS, FGF-2 10 ng/ml and TGFβ_1_10 ng/ml (R&D systems) in monolayer culture until confluence. After 1 passage, the cells were trypsinized from monolayer cultures, counted and divided in polystyrene 15 ml tubes. Each tube containing 5 × 10^5^ cells was centrifuged at 500 g for 10 min at 4°C. The resultant pellets were not resuspended, but cultured in chondrogenic medium for 28 days. The medium was replaced every 3 days and at the end of the culture half of the pellets from each group (Ctr and T-Lys) were collected for histological analysis and the remaining pellets from each group were used for RNA extraction.

### T-LysYal® Treatment

The supramolecular aggregate, T-LysYal® (Sildeha, Switzerland), is patent protected. In the experiments one part of the cells was treated with a concentration of 0.3% v/v T-Lys (treatment group), that was added to the medium at every change (every 3 days). The part of the cells that was not treated with 0.3% v/v T-Lys served as control group (Ctr).

### Real Time RT-PCR

Total RNA was extracted from cells or pellets using RNasy kit (Qiagen, Hilden, Germany) following the manufacturer's instructions. Superscript First-Strand Synthesis System kit (Invitrogen Life Technologies, Carlsbad, CA, USA) was used for the reverse transcription of 2 μg of total RNA. The synthesized cDNA (20 ng) was subjected to quantitative PCR. The analysis of cDNA was performed by a BioRad CFX96 Real Time System using the SYBR green PCR method according to manufacturer's instruction (BioRadiScript Reverse Transcription Supermix cat. 170-8841). The following primer pairs were used for the RT-PCR amplification: sense *Coll I* (COL1A1) 5′-CGTGGCAGTGATGGAAGTG-3′; antisense *Coll I* 5′-AGCAGGACCAGCGTTACC-3′; sense *Runx-2* 5′-GGAATGCCTCTGCTGTTATG-3′; antisense *Runx-2* 5′-TTCTGTCTGTGCCTTCTGG-3′; sense β_2_ *microglobulin* (B2M) 5′-ATGAGTATGCCTGCCGTGTGA-3′; antisense β_2_ *microglobulin* 5′-GGCATCTTCAAACCTCCATG-3′; sense *Sox9* 5′-GCTCTGGAGACTTCTGAACGAGAG-3′; antisense *Sox9* 5′-CGTTCTTCACCGACTTCCTCC-3′; sense *Col II* 5′-CATGAGGGCGCGGTAGAGAC-3′; antisense *Col II* 5′-TGCCAGCCTCCTGGACATC-3′; sense *Aggrecan* 5′- GACTTCCGCTGGTCAGATGG-3′; antisense *Aggrecan* 5′-RCGTTTGTAGGTGGTGGCTGTG-3′. Gene expression was calculated by determining the mean cycle threshold value (Ct) from triplicate samples, normalizing the results to β_2_ *microglobulin* (B2M) levels for each reaction.

### Immunoblotting

Cells were lysed accordingly to protocols previously reported (3, 5). Total protein concentration was assessed using Bio-Rad protein assay kit. The proteins of interest were determined separating equal amounts of proteins for each sample by SDS-PAGE and transferring to nitrocellulose membranes (Invitrogen, Carlsbad, CA). Incubation of membranes with the appropriate primary antibodies, was followed by incubation with IRDye-labeled secondary antibodies (680/800CW) (LI-COR Biosciences, NE), in order to visualize the immune complexes with the Odyssey infrared imaging system (LI-COR Corp., Lincoln, NE).

### Alkaline Phosphatase (ALP)

The levels of ALP, the biochemical marker for the osteoblast activity, were evaluated in DBSCs cultivated in osteogenic conditions, using the kit Leukocyte Alkaline Phosphatase Kit (Sigma Aldrich). Cells were fixed, gently rinsed with deionized water and stained in the dark for 15' with ALP solution, according to manufacturer's instructions. After staining the cells were washed with water, air dried and analyzed with optical microscope. ALP-positive cells appear purple stained.

### Alizarin Red Staining (ARS)

Calcium-rich deposits produced by differentiated DBSCs were detected by Alizarin Red Staining. The cells were gently washed with PBS, fixed with 10% formalin at room temperature for 10 min and then washed again with deionized water to remove fixative residues. ARS solution 1% was added at room temperature for 10 min and discarded. The wells were rinsed twice with deionized water and air dried. The presence of red staining assesses the production of calcium-rich deposits. The dye was extracted from the stained cell layer and assayed for quantification at 405 nm as previously described (20, 23) and the results were evaluated for statistical analysis.

### Immunofluorescence

Cells were cultured on glass coverslips for desired time. At the culture stop, the cells were fixed in 4% (PFA)/PBS, washed with PBS, and blocked in 1% BSA, 5% normal goat serum in PBS for 20 min. Coverslips with fixed cells were incubated with α_V_β_3_ antibody 1:100 (clone LM609 antibody, Millipore, cat. MAB1976). After incubation and washing, bound antibodies were detected using 2 μg/ml of fluorescently labeled goat anti-mouse secondary antibody (Alexa Fluor 488, Invitrogen). Samples were embedded in Mowiol containing 0.1% (v/v) DAPI for an additional staining of the nucleus. The cells were then visualized and photographed using a multispectral confocal microscope Leica TCS SP5.

### Histological Analysis

Pellets were fixed in 4% (PFA)/PBS then processed through graded alcohols and embedded in a cold-curing resin for histological examinations (Technovit 8100—Kulzer) containing 2-hydroxyethyl methacrylate. Sequential sections were cut by a rotary microtome (Reichert-Yung Autocut 1140) into 5 μm for Hematoxylin and Safranin staining. After hydrating sections in the decreasing series of ethyl alcohols up to distilled water and nuclear labeling with Mayer's hemalum solution (Merck),0.1% Safranin O staining was performed to demonstrate the presence of cartilaginous matrix (which stained orange). Microphotographs were captured under optical microscope (Leica) using a 40X objective lens and analyzed using Image-J software.

### Statistical Analyses

Statistical analyses were performed by Student's *t*-test with the Statistical Package for the Social Sciences (spssx/pc) software (SPSS, Chicago, IL, USA). The results were considered statistically significant for *p* < 0.05 (indicated as ^#^*p* < 0.05, ^§^*p* < 0.01, ^*^p < 0.001). ImageJ was used to process images. Western blot bands were quantified using ImageJ.

## Results

### Administration of T-Lys During the Osteogenic Differentiation of MSCs Upregulates the Expression of Osteoblast Markers

**Figure 1:**
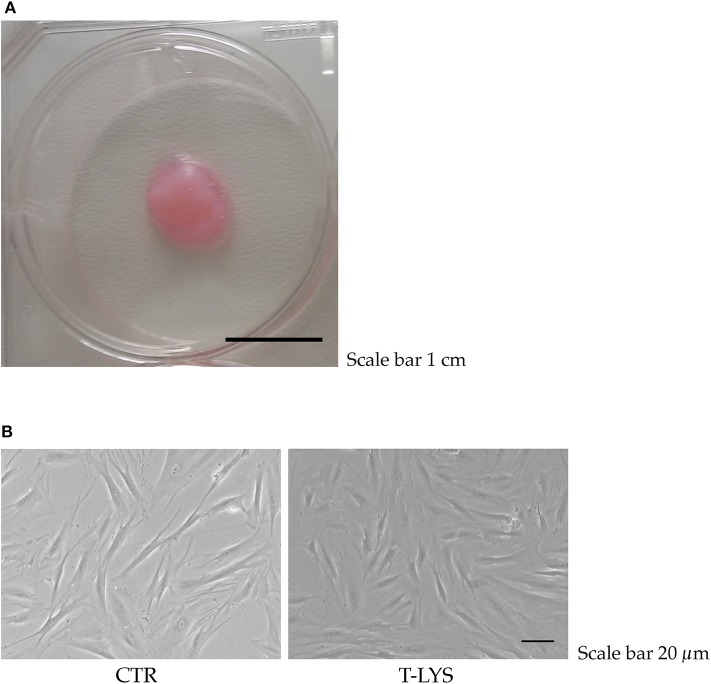
T-Lys treatment in DBSCs cultures. **(A)** Dental bud isolated from a third molar of a healthy patient. Scale bar: 1 cm. **(B)** Representative phase contrast pictures of DBSCs cultivated in osteogenic medium and stimulated with 0.3% v/v T-Lys (T-Lys) or not (Ctr). Scale bar: 20 μm.

We investigated if T-Lys could have an effect in differentiation of MSCs toward the osteoblastic lineage. DBSCs (Figures 1A,B) were used as MSCs and were differentiated for 12 days with osteogenic medium. T-Lys had an evident effect during MSCs differentiation: cells assumed a more osteoblast-like morphology and bone matrix showed a greater mineralized component (Figure 1B).

**Figure 2:**
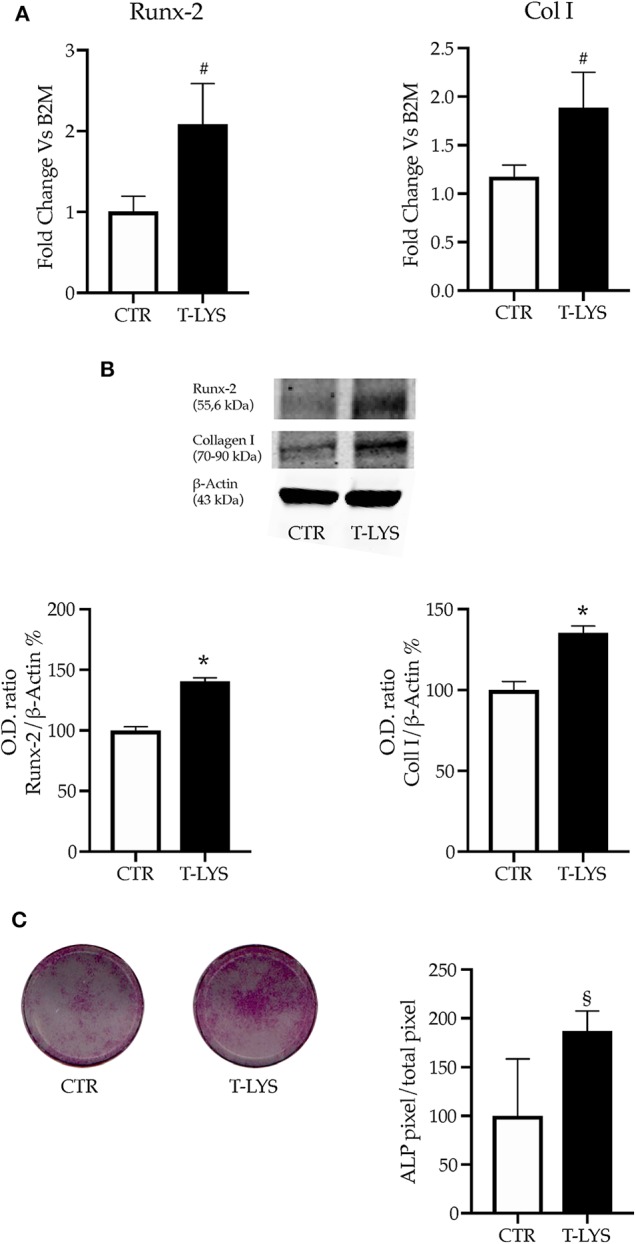
Effects of T-Lys on the expression of osteoblast markers. **(A)** qPCR performed on DBSCs cultivated with osteogenic medium for 12 days and stimulated with 0.3% v/v T-Lys and Ctr. The graphs show that the treatment significantly increased the expression of the two osteoblast markers *Runx-2* and *Col1*. Expression was normalized to β_2_*microglobulin* (B2M). Each graph represents the mean ± SE of 3 independent experiments performed in triplicate. ^#^*P* < 0.05 compared to Ctr. Statistics: unpaired Student's *t*-test. **(B)** Immunoblotting confirmed that the expression of *Runx-2* and *Col1* protein increased in T-Lys treated cells relative to Ctr cells. Each graph represents the mean OD ± SE of 3 independent experiments performed in triplicate. ^*^P < 0.001 compared to Ctr. Statistics: Unpaired Student's *t*-test. Representative immunoblots were chosen for the figure. **(C)** ALP histochemical assay (purple staining) performed on DBSCs maintained in osteogenic conditions for 7 days and stimulated with T-Lys and Ctr. The graph represents the quantification of positive staining as percentage compared to Ctr (^§^*P* < 0.01) and is representative for 3 independent experiments performed in quadruplicates. Data are presented as mean ± SE. Student's *t*-test was used for single comparisons. The wells of a representative experiment were chosen for the figure.

The mRNA levels of the typical osteoblast early markers, *Runx-2* and *Collagen I* (*Col1*), were determined in Ctr and T-Lys treated samples by using real time PCR, after 12 days of osteogenic differentiation. Interestingly, the Figure 2A shows that the expression of both markers significantly increased in T-Lys treated cells compared to Ctr cells, indicating that T-Lys treatment enhanced the capacity of MSCs to commit to osteoblast lineage. The protein expression levels of such osteoblastic markers were further evaluated in T-Lys treated and Ctr cells by western blot analysis. In Figure 2B is reported that *Runx-2* protein level raised, as did *Col1* protein level, in T-Lys treated cells in comparison to Ctr cells thus confirming the mRNA expression trend. Subsequently a histochemical test was performed to explore the expression of another marker of osteoblast cells, the enzyme Alkaline Phosphatase (ALP), in response of T-Lys treatment. The result of this experiment, showed in the Figure 2C, revealed that MSCs stimulation with T-Lys, during 7 days of osteogenic differentiation, significantly increased the purple staining identifying ALP expression. These findings, resembling the expression trend of *Runx-2* and *Col1*, demonstrated that T-Lys is capable to increase the ability of MSCs to differentiate in osteoblast-like cells and supported the molecular pattern results with a phenotypical characteristic.

### T-Lys Treatment Increases the Amount of Mineral Matrix Deposition During Osteogenic Differentiation of MSCs

**Figure 3:**
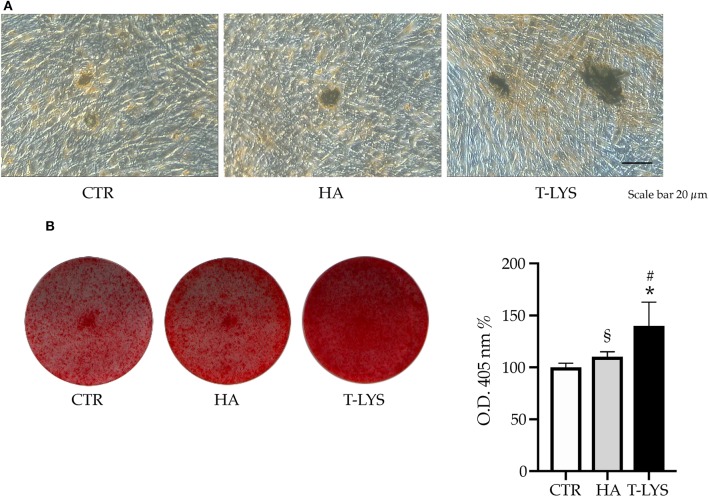
Mineral matrix deposition following T-Lys stimulation and Alizarin Red quantification. **(A)** Formation of calcium rich deposits in representative phase contrast pictures of DBSCs treated with T-Lys, HA or Ctr for 21 days in osteogenic conditions. Scale bar: 20 μm. **(B)** Mineral matrix deposition assayed by ARS (red staining). The graph shows the OD quantification of extracted dye from stained cell layers as mean percentage ± SE and is representative for 3 independent experiments performed in quadruplicates. ^§^*P* < 0.01, ^*^P < 0.001 compared to Ctr; ^§^*P* < 0.01 compared to HA. Student's *t*-test was used for single comparisons. The wells of a representative experiment were chosen for the figure.

In order to deeply investigate the effect of this new molecule on the osteogenic differentiation of MSCs, we performed DBSCs culture in mineralizing conditions for 21 days and treated the cells with 0.3% v/v T-Lys, that was added to the culture on every medium change. We used, as positive control, hyaluronic acid and the results showed a stronger effect of T-Lys. By using the histochemical assay Alizarin Red Staining (ARS) we analyzed the effect of T-Lys on mineral matrix deposition of DBSCs. ARS was then quantified with a colorimetric technique. The Figure 3 shows that the mineralization capacity of the cells treated with 0.3% v/v of T-Lys was significantly increased compared to the Ctr. This finding, taken together with the results obtained for the osteoblast markers *Runx-2, Col1* and ALP, demonstrated that T-Lys was able to increase the osteogenic capacity of MSCs and stimulate their ability to produce mineralized matrix.

### T-Lys Treatment Affects α_V_β_3_ Subcellular Distribution

**Figure 4:**
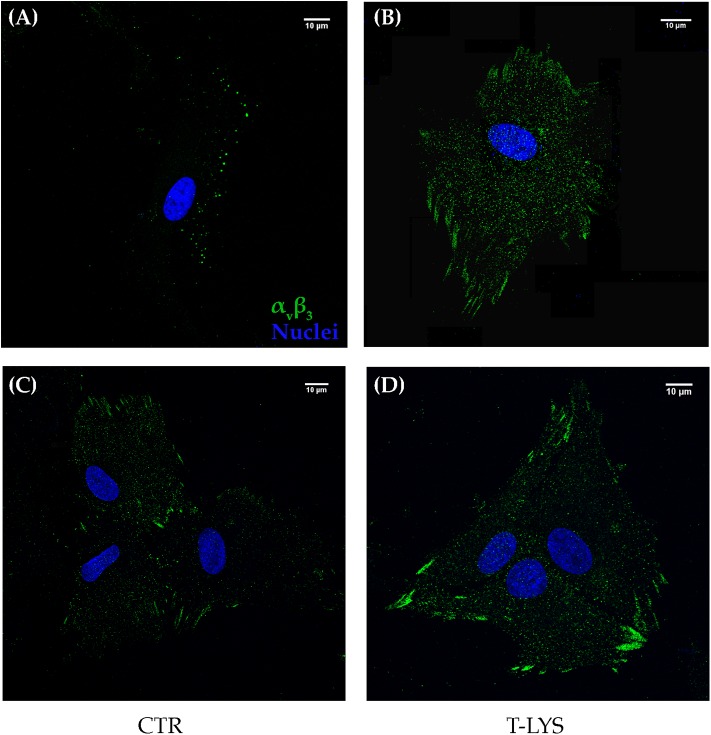
Subcellular localization of integrin α_V_β_3._ Midsection confocal microscopy images show the expression of integrin α_V_β_3_ (green) detected by the antibody LM609 in cells maintained for 4 days in osteogenic medium and treated with T-Lys and Ctr; picture **(A)** shows a single cell with integrin distributed in multiple pointed sites in Ctr, while T-Lys treatment induced a different organization of the integrin that appeared strongly augmented and more clustered and localized at the focal adhesion sites **(B)**. Similar distribution is observable in clustered cells **(C,D)**. Images of a representative experiment were chosen for the figure. Scale bar: 10 μm.

Integrins are receptor for ECM molecules, important in cells adhesion, but also in mediating proliferation and differentiation signals. For this reason, we wandered if a derivative of HA, a component of ECM of connective tissues, could in some way influence a member of Integrin superfamily. To this purpose, we analyzed by confocal immunofluorescence, if T-Lys treatment could affect the subcellular distribution of integrin α_V_β_3_. This Integrin is the receptor for the bone matrix protein Osteopontin, among others, and we showed in a previous work that is fundamental for osteogenic commitment of MSCs (3). The subcellular distribution of integrin α_V_β_3_ was analyzed by confocal microscopy in DBSCs treated with T-Lys and Ctr. Analyses were performed, after 4 days of osteogenic differentiation, due to the rapid propensity of the cells to form a multilayer preventing their microscopic observation. As shown in the Figure 4, in Ctr cells the integrin α_V_β_3_ was distributed in multiple sites, while T-Lys treatment induced a different integrin organization that appeared to be more clustered and localized at the focal adhesion sites. In summary, after 4 days of differentiation, the receptor in control conditions was still distributed throughout the cell, but in the cells treated with T-Lys, it was present in focal adhesions (FAs). The presence of strings (the typical pattern of α_V_β_3_ involved in FAs) was visible in T-Lys treated cells if compared with untreated cells. These results suggested that the effect of T-Lys on DBSCs differentiation could be mediated by α_V_β_3_ rearrangement.

### Administration of T-Lys to Chondrocyte Pellet Cultures Upregulates the Expression of Typical Markers

**Figure 5:**
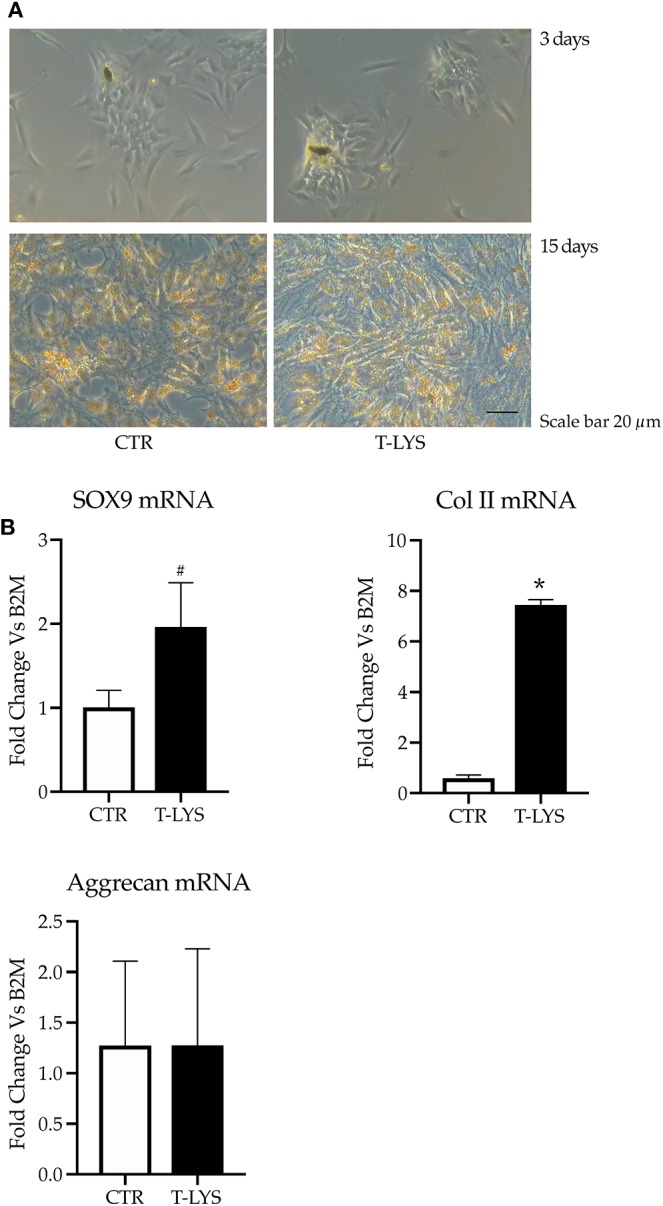
Effects of T-Lys on the expression of chondrocyte markers. **(A)** Representative phase contrast pictures of HACs treated with T-Lys (T-Lys) or not (Ctr) after 3 days (upper panel) and 15 days (lower panel) of culture. T-Lys treatment significantly increased matrix deposition at the expense of a dedifferentiation process after 15 days of culture (lower panel). Scale bar: 20 μm. **(B)** qPCR performed on chondrocyte pellet cultures cultivated 28 days with chondrogenic medium and stimulated with 0.3% v/v T-Lys and Ctr. The graphs show that the treatment significantly increased the expression of the chondrocyte markers *Sox-9* and *Col II*, while had no effect on the expression of Aggrecan. Expression was normalized to β_2_*microglobulin* (B2M). Each graph represents the mean ± SE of 3 independent experiments performed in triplicate. ^#^*P* < 0.05, ^*^P < 0.001 compared to Ctr. Statistics: unpaired Student's *t*-test.

We performed chondrocyte 2D cultures using HACs, harvested from patients who had undergone orthopedic surgery and, according to literature (22), we found chondrocyte-like cells in short term cultures that appeared to respond positively to T-Lys presence (Figure 5A upper panel). Long term cultures, although showing a greater matrix production with T-Lys treatment, seemed to dedifferentiate assuming a fibroblast-like morphology (Figure 5A lower panel). Subsequently, HACs were grown in pellet cultures in order to mimic the three-dimensional tissue micro architecture and avoid the improper chondrocyte dedifferentiation that easily occurs when cultured in two dimensions. The cell pellets were grown for 28 days in chondrogenic conditions; at the same time, they were treated with 0.3% v/v T-Lys, that was added at the cultures on every medium change. At the end of the culture period, the pellets were lysed and assessed for genes expression analysis. The mRNA levels of the typical chondrogenic markers, *Sox-9, Collagen II* (*Col II*) and *Aggrecan* were determined in T-Lys treated and Ctr samples by using real time PCR. Interestingly, the Figure 5B shows that the expression of *Sox-9*, which is the main transcription factor involved in chondrogenic differentiation, significantly increased in T-Lys treated cells, compared to Ctr cells. In agreement with this result, *Col II*, a typical protein of the cartilage extracellular matrix was increased following T-Lys treatment indicating that this molecule supports and enhances chondrocyte differentiation, while the expression of *Aggrecan*, a cartilage proteoglycan, was not influenced.

### T-Lys Stimulates Chondrocytes Number and Tissue Growth

**Figure 6:**
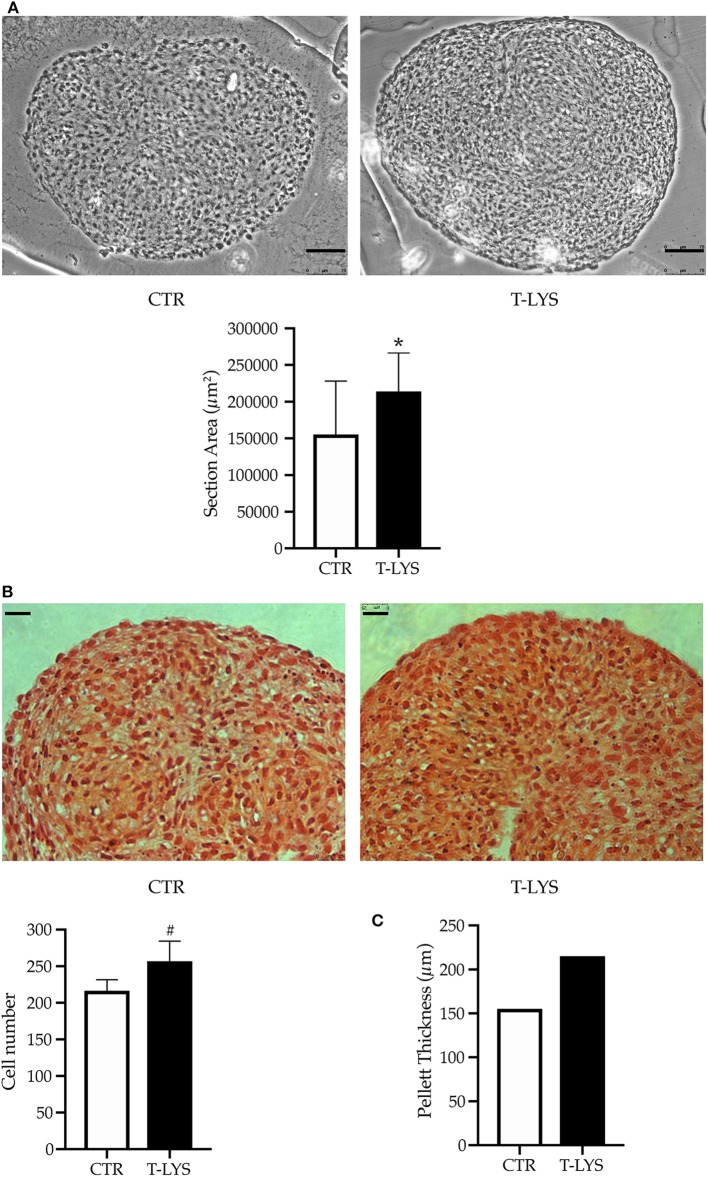
Effects of T-Lys on chondrocytes proliferation and tissue growth. **(A)** Sectioned chondrocyte pellets were captured under optical microscope using a 20X objective lens and analyzed by using Image-J software for morphometric examination of the areas. T-Lys pellet appeared larger than Ctrs. The pictures selected are representative of three different experiments, scale bar: 75 μm. The graph represents the mean ± SE of 3 independent experiments performed in triplicate, ^*^P < 0.001. **(B)** Deposition of cartilaginous matrix was demonstrated by Safranin O staining and chondrocyte nuclei were counterstained with hematoxylin. The pictures were captured with 40X objective lens, scale bar: 25 μm. The graph represents the mean ± SE of 3 independent experiments performed in triplicate, ^#^*P* < 0.05. **(C)** The graph shows a theoretical reconstruction of the pellet thickness where the number of sections obtained was multiplied by the slice thickness (μm).

After 28 days of differentiation, in the conditions described in the previous paragraph, chondrocytes pellets were fixed with 4% PFA, embedded, sectioned and subjected to histological staining and examination. Morphometric examination by optical microscope of sectioned pellets revealed that T-Lys pellets were larger than Ctr ones (Figure 6A). To quantify the pellet dimensions, the samples were sectioned (5 μm thickness) and the area of each section, obtained from T-Lys and Ctr, was measured by ImageJ software. The graph in the Figure 6A shows that the surface average was significantly larger in T-Lys vs. Ctr.

Pellets were stained with Safranin to highlight chondrocytes (samples for T-Lys and Ctr are shown in Figure 6B). Safranin O staining revealed the presence of cartilaginous matrix (orange staining), demonstrating that cells were able to differentiate and produce the components of ECM in these culture conditions; nuclei were counterstained with hematoxylin (Figure 6B). To verify if the T-Lys treatment had also an influence on the cell number, we counted the cells in a selected field (100 × 100 μm) for each section. As indicated in the graph of Figure 6B, cell number was significantly increased in T-Lys pellets compared to Ctr. Interestingly, also a higher number of slices was obtained from T-Lys compared to Ctr. This difference is represented in the graph in Figure 6D, where the number of slices was multiplied by the slice thickness (5 μm) giving a theoretical reconstruction of the pellet thickness. These results demonstrate that T-Lys stimulates chondrocytes proliferation, their differentiation and matrix secretion.

## Discussion

Tissue regeneration, that hopefully occurs ensuring healing after injury or diseases, is a process based on cell renewal and differentiation: specifically bone and cartilage differentiation profits by osteogenic and chondrogenic differentiation. These two processes, as well as for all the connective tissues, start from MSCs differentiation. Stem cell differentiation is affected by the micro-environment: the ECM governs maintenance and propagation of MSCs (24) and it contains macromolecules such as collagen, adhesive glycoprotein, and GAG. HA, which is a ubiquitarian GAG, has been demonstrated to stimulate MSCs differentiation by affecting the ECM composition (25).

In this work we demonstrated, for the first time, the impact of an innovative molecule, T-Lys, on MSCs osteogenic and chondrogenic differentiation. T-Lys had an impressive effect because, comparing its structure to the one of the HA, it is a more stable but dynamic molecule. The aim of the work was to study the effects of T-Lys in both the supportive connective tissues: bone and cartilage. However, the research findings concerning MSCs differentiation prompted us to standardize experimental protocols using T-Lys in MSCs osteogenic differentiation. On the other hand, since an ideal protocol for chondrogenic differentiation of MSCs is still challenging (17), we used chondrocytes isolated from young patient articular cartilage following the indications in literature related to chondrogenesis (18). Culture conditions were thus calibrated for osteogenesis and chondrogenesis opting for three-dimensional culture for the latter.

Using DBSCs as MSCs source, we analyzed the expression of typical osteoblastic markers during cell differentiation process into the osteogenic lineage in the presence or not of T-Lys. We demonstrated that this supramolecular aggregate is able to enhance the acquisition of the osteogenic features in DBSCs. We found that T-Lys treatment significantly promoted the expression of *Runx-2* and *Col1* at both mRNA and protein level (Figures 2A,B). These data are correlated with the induced ALP activity on day 7 and the formation of calcium rich deposits at 21 days of differentiation (Figure 2C). This positive effect was remarkably stronger than the one detected when HA was used (Figure 3).

Moreover, considering that cell adhesion mechanisms impact on cell differentiation (26, 27) and in particular α_V_β_3_ integrin is crucial in the commitment of MSCs into osteoblast lineage (3), we examined this integrin expression in DBSCs treated with T-Lys. We detected that the treatment determined a higher clusterization of the receptor into FAs, if compared to the untreated cells (Figure 4), suggesting that the effect of T-Lys on integrin organization drives in turn DBSCs differentiation. All these observations demonstrated that DBSCs respond to T-Lys treatment and are also consistent with the data in literature which attribute to HA a role in inducing osteogenic differentiation of MSCs from human bone marrow (24, 28). We also investigated how T-Lys influenced chondrogenic differentiation process in chondrocyte tridimensional cultures. We observed that the HA derivative induced an increased expression of typical chondrogenic markers, such as the main transcription factor *Sox-9*, and the principal collagenous protein of the cartilage extracellular matrix *Col II*, if compared with control chondrocyte cultures (Figure 5B). The molecular results correlated with morphological data; thus we found that the surface average of sectioned pellets was significantly larger in T-Lys vs. Ctr (Figure 6A) and the cell number was significantly increased (Figure 6B). Interestingly we obtained a higher number of sections from T-Lys pellets compared to Ctr and we predicted a theoretical reconstruction of the pellet thickness, multiplying the number of slices by the slice thickness (Figure 6C). These results demonstrate that T-Lys stimulates chondrocytes proliferation, differentiation and tissue growth; indeed, Safranin O staining revealed the presence of cartilaginous matrix secretion (orange staining), indicating that cells compacted in pellets were able to differentiate and produce the components of ECM. This finding is in agreement with previous data, indicating that HA influences articular chondrocytes proliferation and ECM production (29, 30).

The weakness of this study concerns the two different cell models used to elucidate T-Lys effect: this investigation method has been conditioned by the choice of the most trustable protocols scientifically approved for osteoblast and chondrocyte differentiation. The strength of this work is the demonstration that T-Lys has a remarkable effect in both bone and cartilage, administering the molecule to undifferentiated cells; this gave the opportunity to highlight T-Lys effects during cell differentiation, showing a notable influence on the activity of osteoblasts and chondrocytes and on matrix deposition.

Research studies and clinical applications demonstrate that hard tissue regeneration is supported by the presence of scaffolds (31, 32). Cell engraftment, proliferation and differentiation in the lesion subjected to regeneration also depend on the scaffold features. HA has been widely investigated and used for various applications in association with scaffold because of its peculiar characteristics. HA is present in cartilage both as single molecule and complexed in proteoglycan polymers; HA gives to the matrix its peculiar features because of chemical and physical properties. Cartilage tissue engineering should support cell growth and differentiation, but the process needs to start from cell adhesion. HA has been demonstrated to develop weak cell adhesion even when integrated in the opportune scaffold i.e. PEG (polyethylene glycol) (33).

The chemical structure of T-Lys has some special features. Firstly, it is known that in T-Lys lysine hyaluronate and thymine are located at the target sites of the lytic enzyme hyaluronidase, making the molecule extremely stable and more resistant to degradation. Besides, because of the molecular conformation, T-Lys not only binds to water, as HA, but in addition is able to move it in the tissue, thus modifying the cell microenvironment most likely favoring cell adhesion, differentiation and secretion. These features make the molecule more stable, determining a more effective repair action.

T-Lys, as a HA derivative, offers a superior stability and prolonged effects; in this context we made a step forward identifying in this new aggregate a molecule that enhances osteogenic and chondrogenic differentiation processes. The results of this study suggest that the integration of the opportune scaffolds with T-Lys could be considered a promising optimization for both cartilage and bone regeneration.

## Data Availability Statement

The raw data supporting the conclusions of this article will be made available by the authors, without undue reservation, to any qualified researcher.

## Ethics Statement

The studies involving human participants were reviewed and approved by ethical committee of the University of Foggia Ospedali Riuniti, within the project BIADIDEBT num. Rep 4159/2018. Written informed consent to participate in this study was provided by the participants' legal guardian/next of kin. Written informed consent was obtained from the individual(s) for the publication of any potentially identifiable images or data included in this article.

## Author Contributions

GM designed the study and prepared the first draft of the paper and guarantor. AD, FP, and MC contributed to the experimental work. AD and FP were responsible for statistical analysis of the data. LL, EC-A and GM supervision. GM and FG Resources. MM, RG, and ZK methodology. All authors revised the paper critically for intellectual content and approved the final version. All authors agree to be accountable for the work and to ensure that any questions relating to the accuracy and integrity of the paper are investigated and properly resolved.

## Conflict of Interest

The authors declare that the research was conducted in the absence of any commercial or financial relationships that could be construed as a potential conflict of interest.
